# Application of Pre-Wetted High Titanium Heavy Slag Aggregate in Cement Concrete

**DOI:** 10.3390/ma15030831

**Published:** 2022-01-22

**Authors:** Tao Zhang, Bei Huang

**Affiliations:** 1 College of Materials Science and Engineering, Nanjing Tech University, Nanjing 211800, China; 201961203162@njtech.edu.cn; 2 State Key Laboratory of Materials-Oriented Chemical Engineering, Nanjing 211800, China

**Keywords:** high titanium heavy slag, concrete, strength, shrinkage

## Abstract

High titanium heavy slag is one kind of solid waste that exists in large amounts in the southwest of China. In this paper, this high titanium heavy slag is used as natural pre-wetted material in concrete because of its porous structure. Three kinds of aggregates are used in this concrete. The first one is natural limestone and river sand. The second one is dry slag fine aggregate and coarse aggregate. The third one is pre-wetted coarse slag aggregate and dry slag fine aggregate. The strength, dry shrinkage, autogenous shrinkage, relative humidity, pore size distribution, stress–strain relationship, micro-hardness and chloride penetration of concrete composed of the above three aggregates are tested in this study. The results show that pre-wetted slag aggregate is a suitable internal curing material. The concrete with pre-wetted slag aggregate shows higher strength, lower shrinkage and smaller porosity. The water absorbed in the slag aggregate can be released effectively to increase the relative humidity, accelerate hydration, improve porosity and increase the interface strength.

## 1. Introduction

High titanium heavy slag is the solid waste product after smelting vanadium titanium magnetite, which exists in large quantities in Guizhou Province in China. This high titanium heavy slag has a porous structure with no harmful ingredients [1]. This slag could be used as an aggregate in concrete, and the workability of concrete with this slag aggregate could be adjusted by chemical admixture [2] to fit the pumping requirement in the construction.

The volume stability is a matter of concern for the durability of concrete structures. In order to enhance the volume stability of concrete, several methods are used. Firstly, the volume deformation of concrete is controlled by adding shrinkage-reducing admixture [3,4,5,6,7]. The study results show that the dry shrinkage declines and the pore size decreases by using shrinkage-reducing admixtures. However, the adaptability of chemical admixture and components in concrete is a key problem to which attention should be paid. Additionally, the high price of this admixture hinders its wide use in concrete. Secondly, different kinds of fibers [8,9,10] and expansive agents, such as MgO [11,12,13,14], are used to improve the volume stability of concrete too. Both expansive agents and fibers can improve the volume stability effectively, but the workability of concrete with these materials is adjusted through the other admixture, which will increase the cost of the concrete. Thirdly, internal curing technology is a useful method to maintain the volume stability of concrete. In some studies, a light aggregate is used as pre-wetted material to improve the shrinkage of cementitious material. However, the strength of the sample would decrease when the concrete is composed of a great deal of lightweight aggregate [15,16,17,18,19]. Therefore, this method is not suitable for casting high-strength concrete.

Aggregate in concrete occupies 60 to 80 percent of the volume, so it plays a major role in concrete. High titanium heavy slag is a natural porous aggregate, and its mechanical properties are similar to natural gravel. If it can be used as internal curing material, it can not only improve the volume deformation of concrete but also not increase the cost of concrete. Therefore, in this paper, the feasibility of this problem is discussed.

## 2. Research Significance

High titanium heavy slag is solid waste with no harmful components. Originally, this waste was used as foundation filling material. In this paper, the feasibility of the use of this slag as an aggregate in concrete is studied. Furthermore, slag has a porous structure and higher crushed indexes than light porous aggregate. Therefore, the suitability of the use of this slag as an internal curing agent in concrete is discussed firstly.

## 3. Experimental

### 3.1. Aggregates

**Figure 1:**
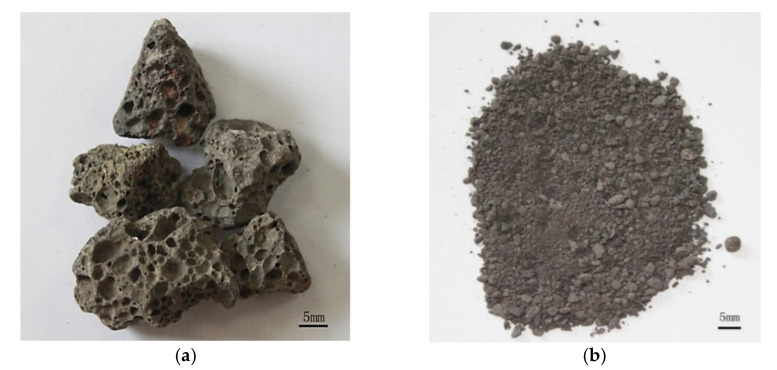
Appearance of high titanium heavy slag. (**a**) coarse aggregate, (**b**) fine aggregate.

**Figure 2:**
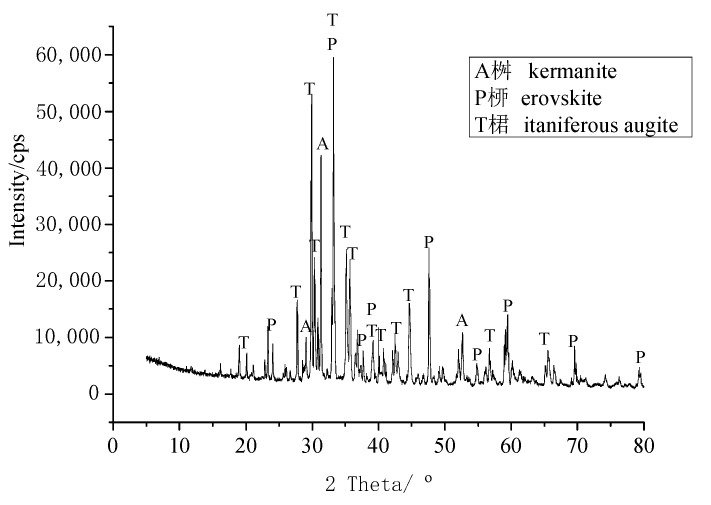
XRD pattern of slag aggregate.

Two kinds of aggregates are used in this study. One is the high titanium heavy slag, which is crushed into fine aggregate (≤4.75 mm) and coarse aggregate (5–20 mm), from Guizhou Province. Another one is limestone and river sand. The appearance of slag aggregate with high content of titanium is shown in Figure 1, and its chemical compositions are shown in Table 1. Slag is grounded into powder and the XRD data are collected in the range of 5°–80°, 2θ at a counting time of 15 s/step and a divergence slit of 1°. The mineral compositions of slag are shown in Figure 2. In Figure 1, the slag aggregate porous structure is presented, which provided the ability to absorb water within the aggregates. The crush indexes, apparent density and water content in saturated state of slag aggregates, limestone (5–20 mm) and river sand (≤4.75 mm) were tested by Chinese national standard GB/T14684-2011, GB/T14685-2011, and the results are displayed in Table 2.

### 3.2. Cement and Fly Ash

The concrete is composed of aggregate, fly ash and P·O 42.5 ordinary Portland cement, which meets the Chinese standard GB 175-2007. The chemical compositions of cement and fly ash are shown in Table 3.

### 3.3. Mix Proportion of Concrete

In our experimental program, three kinds of concrete are molded. First one is natural aggregates concrete (NAC), including limestone and river sand. Second one is dry slag aggregates concrete (DSAC) containing dry slag fine aggregate and coarse aggregate. The third one is pre-wetted slag aggregate concrete (WSAC) with coarse slag aggregate and dry slag fine aggregate. The mix proportions of concrete with these three different types of aggregate are shown in Table 4. In WSAC group, the coarse slag aggregate is soaked into the water for 24 h in advance and then covered with the wetting cloth to keep saturated surface dry state before casting.

### 3.4. Test Methods

#### 3.4.1. Mechanical Properties

**Figure 3:**
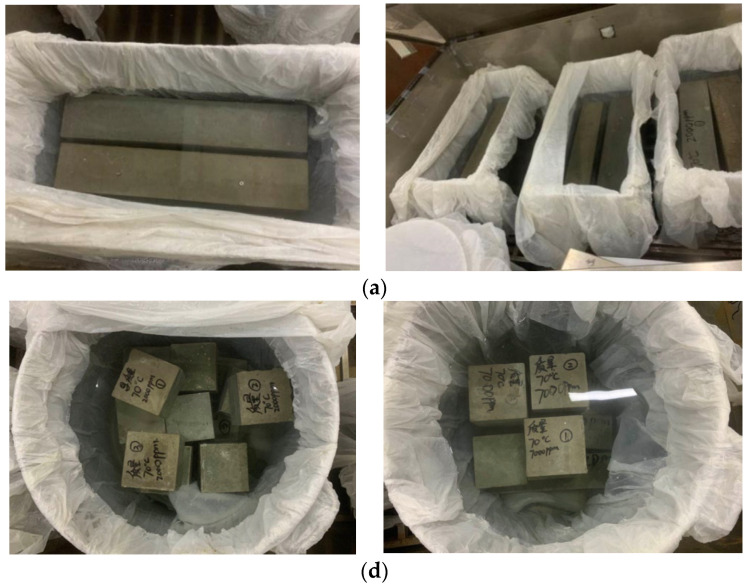
Samples to mechanical testing. (**a**) sample for flexural tensile strength test, (**b**) sample for compressive strength and splitting tensile strength test.

**Figure 4:**
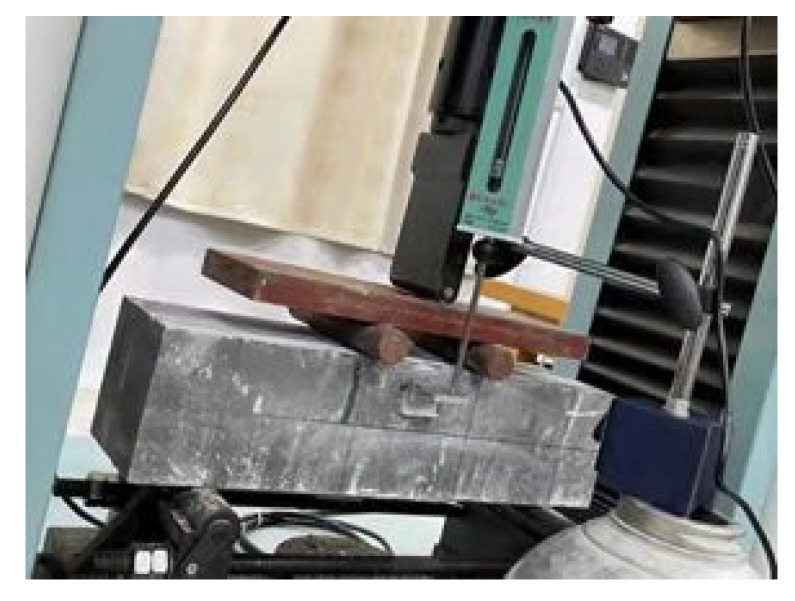
Device of flexural strength testing.

The three species of samples are demolded a day after casting and cured in a fog room (20 ± 2 °C, 95% relative humidity) for different curing ages. The samples with the size of 100 × 100 × 100 mm are used to cubic compressive strength and splitting tensile strength testing. The 150 ×150 × 550 mm prism specimens are used to flexural strength, as shown in Figure 3 and Figure 4. The specimens with the size of 70 × 70 ×210 mm are used to get stress and strain relationship of concrete composed with different groups of aggregates. All the tests are practiced according to Chinese national standard GB/T50081-2019. The loading speeds of compressive strength, splitting tensile strength and flexural tensile strength are 0.5MPa/s-0.8MPa/s, 0.05MPa/s-0.08MPa/s and 0.05MPa/s-0.08MPa/s, respectively, according to GB/T50081-2019.

#### 3.4.2. Dry Shrinkage Testing

**Figure 5:**
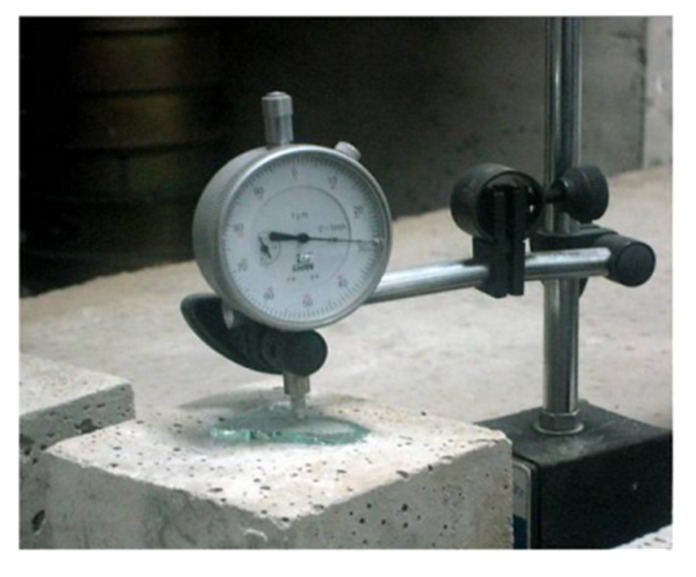
Drying shrinkage testing of concrete.

The prism concrete specimen with size of 150 × 150 × 550 mm is formed, and it is cured for 28 days before being put into the shrinkage testing room, where the temperature is kept at 23 ± 2 °C and humidity is about 50 ± 3% according to GB/T 29417-2012. The shrinkage deformations of specimens are measured by dial gauge, as presented in Figure 5. A piece of glass is put on the surface of the sample to keep testing surface smooth.

#### 3.4.3. Autogenous Shrinkage and Internal Humidity Measurement

**Figure 6:**
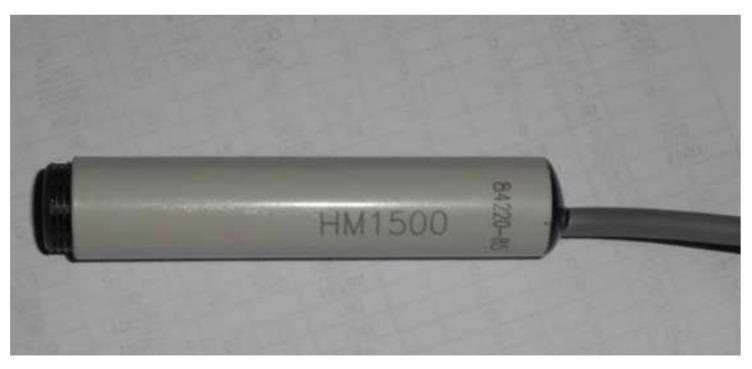
Moisture meter embedded in concrete.

**Figure 7:**
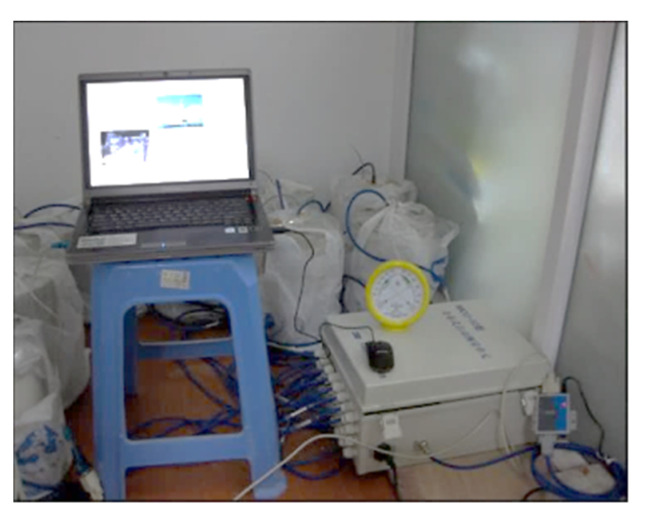
Autogenous shrinkage and internal humidity testing.

The cylinder concrete specimens of 100 mm in diameter and 200 mm in length are casted in PVC tube, which are used to obtain the autogenous shrinkage deformation and relative humidity in concrete up to 125 days according to SL/T 352-2020. The whole cylinder sample is coated by the insulation film to isolate the temperature and humidity exchange between samples and environment, and the vibration-wire strain gauge and the moisture meter (shown in Figure 6) are both embedded in each specimen to measure the autogenous shrinkage and humidity variation inside the concrete. All the experimental data are collected by data acquisition device automatically, as displayed in Figure 7.

#### 3.4.4. MIP

In order to analyze the porosity of samples, the specimens curing for 120 d are subjected to measurements of pore volume and pore size distribution using intrusion porosimetery (MIP, Auto Pore 9510, Poremaster gt-60, quantachrome, USA).

#### 3.4.5. Failure Form under Uniaxial Compressive Strength

**Figure 8:**
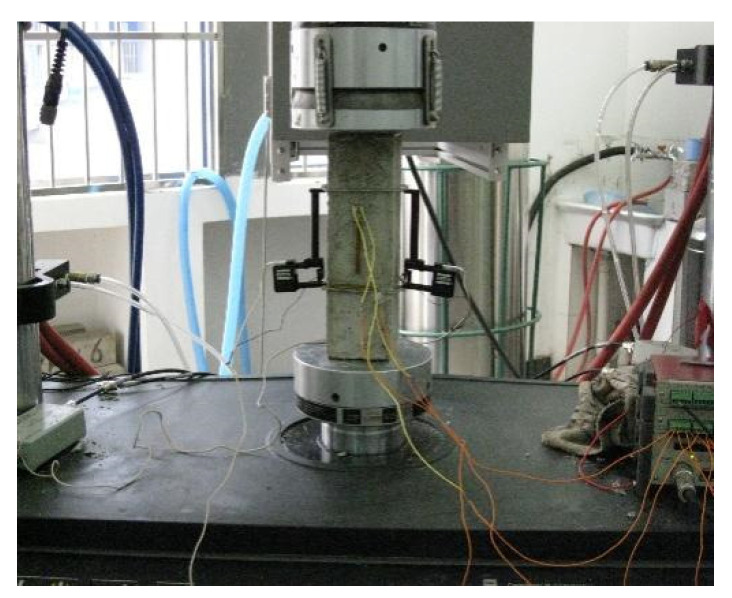
Sample and loading setup.

The samples are taken out after curing for 28 days. The stress and strain relationships under uniaxial compressive loading of concrete made of different groups of aggregate are tested by 300 KN material test system (MTS, American Instron 300 KN material testing machine). The axial deformation is measured by LDVT (American Instron 300 KN material testing machine), as shown in Figure 8. The loading speed is controlled at 0.2 mm/min [20].

#### 3.4.6. Chloride Penetration Test

**Figure 9:**
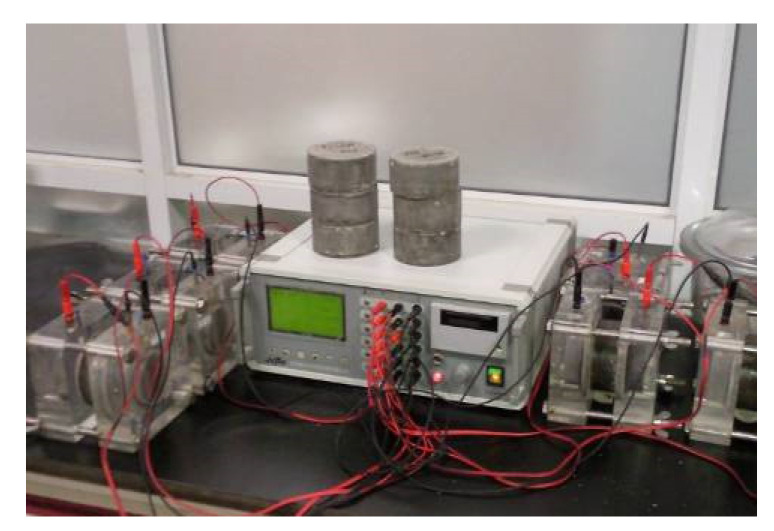
Electric flux meter.

In order to analyze the compactness of the samples, the concrete cores of φ100 × 50 mm are drilled out from 150 × 150 × 150 mm cubic samples, which consist of three various kinds of aggregate, at 7d and 28d. Then, each core sample is cut into three parts with the same size of φ100 × 50 mm. The cutting samples are subjected to chloride penetration test according to ASTM C1202 and the relative device, as shown in Figure 9.

#### 3.4.7. Micro-Hardness Test

In order to study the interface strength between aggregate and paste in concrete consisting of different types of aggregates, the concrete sample is cut and the cross section is polished. The average micro-hardness values in interface transition zone and paste matrix are at least measured at 10 points around each aggregate and paste zone, respectively.

## 4. Results

### 4.1. Mechanical Properties

**Figure 10:**
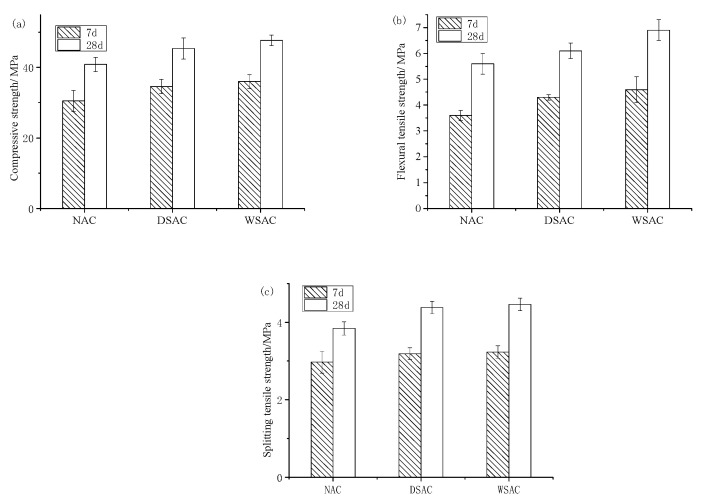
Strength of NAC, DSAC and WSAC samples at 7d and 28d. (**a**) compressive strength. (**b**) flexural tensile strength. (**c**) splitting tensile strength.

The compressive strength, flexural strength and splitting tensile strength of six samples with each kind of mix proportion were tested. The average values of strength of NAC, DSAC and WSAC samples at 7 days and 28 days are shown in Figure 10. Figure 10 presents that, with the development of the curing time, all three strengths of each group of specimens increased noticeably. Compared with these three series, the concrete with pre-wetted coarse slag aggregate and dry slag sand showed the highest strength, and the concrete made of natural limestone and river sand showed the minimum strength value.

### 4.2. Dry Shrinkage

**Figure 11:**
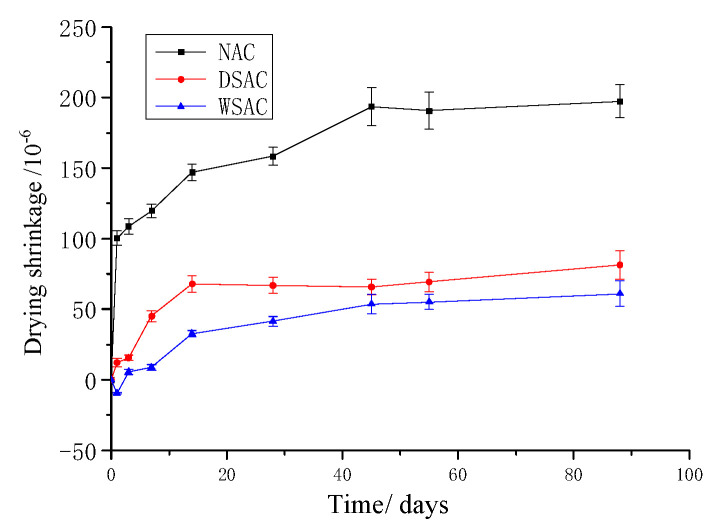
Dry shrinkage development of samples up to 90d.

Three samples with each mix were used for the dry shrinkage measurement. Figure 11 presents the average dry shrinkage of the NAC, DSAC and WSAC samples up to 90 days. From Figure 11, it is shown that the shrinkage variation trends of concrete specimens with slag aggregate are similar. The dry shrinkage of both the DSAC and WSAC samples increase significantly before 14 days, and then the shrinkages of the samples change a little after 20 days. However, for the NAC sample, the dry shrinkage value increases obviously before 45 days, and the shrinkage deformation keeps constant after 45 days. The maximum deformation of dry shrinkage of NAC, DSAC and WSAC specimens are 197 μm, 82 μm and 61 μm respectively. Thus, the concrete with slag aggregate can decrease the dry shrinkage significantly compared with the one with natural aggregate. It is illustrated that the high titanium heavy slag aggregate can decrease the dry shrinkage effectively.

### 4.3. Autogenous Shrinkage

**Figure 12:**
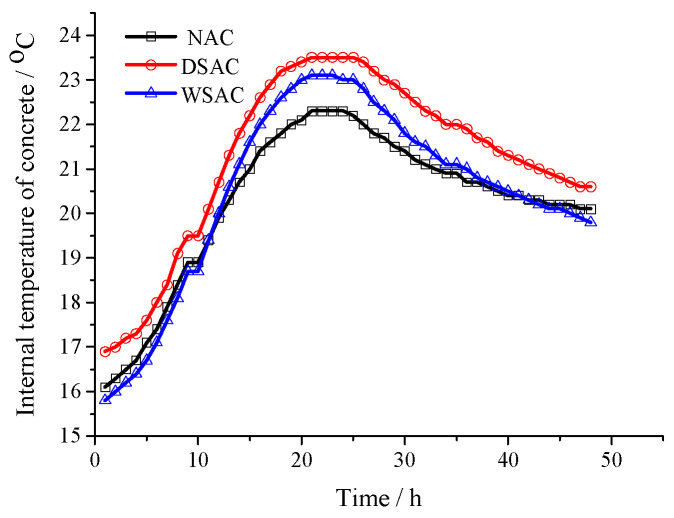
Temperature change in the concrete.

**Figure 13:**
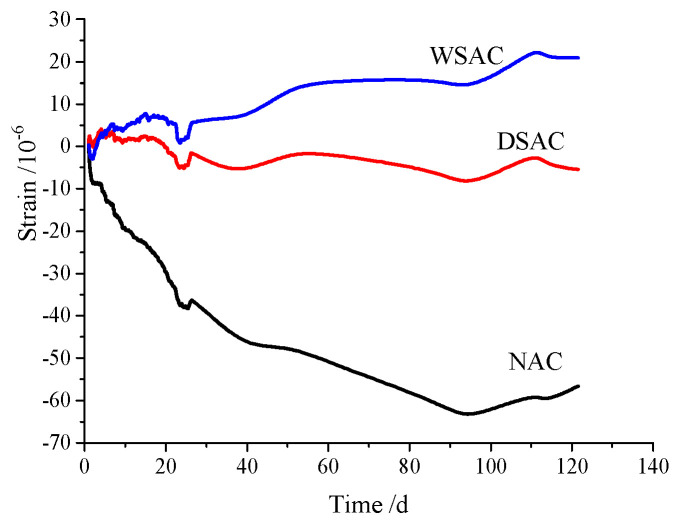
Autogenous shrinkage of concrete.

The temperature change and autogenous shrinkage in the concrete samples tested by vibration-wire strain gauge embedded in concrete are presented in Figure 12 and Figure 13, respectively. Figure 12 indicates that the interior temperature of each concrete sample ranged from 16 °C to 23 °C because of the cementitious materials content in all the concrete mix proportions, as shown in Table 4. In Figure 13, it is suggested that the sample with natural limestone and sand presents apparent volume shrinkage during the time. The volume shrinkage of the specimen made of dry slag aggregate is not obvious, and the samples with pre-wetted coarse slag aggregate and dry fine slag aggregate expanded clearly up to 125 days.

### 4.4. Internal Relative Humidity

**Figure 14:**
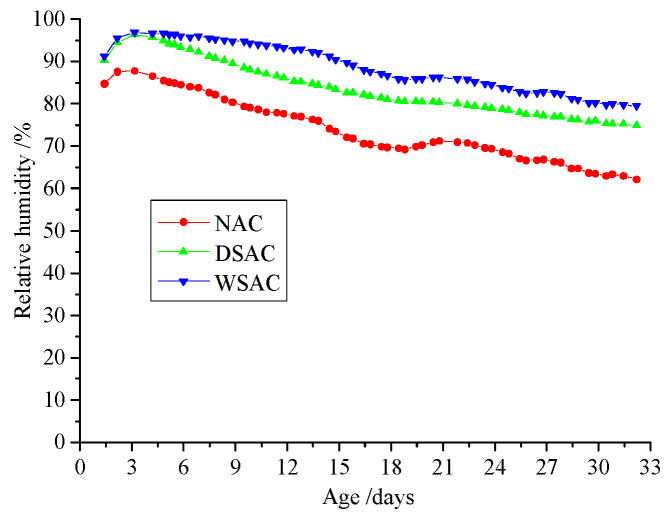
Relative humidity in concrete.

Figure 14 illustrates the relative humidity in concrete during autogenous shrinkage measurement. It is shown that, during the hydration, the relative humidity in the concrete decreased, and the RH values of the NAC, DSAC and WSAC samples were 62%, 74% and 80%, respectively. Obviously, the relative humidity in the specimen with pre-wetted coarse slag aggregate was higher than the other groups as the water pre-absorbed in the coarse porous aggregate could be released when the environmental humidity decreased. The humidity of the concrete containing dry slag aggregate was a little lower than that of the WSAC sample. Because of the porous structure of slag aggregate, the water can uptake into the aggregate in the process of casting, and this part of water can also increase the humidity during the hydration.

### 4.5. MIP Analysis

**Figure 15:**
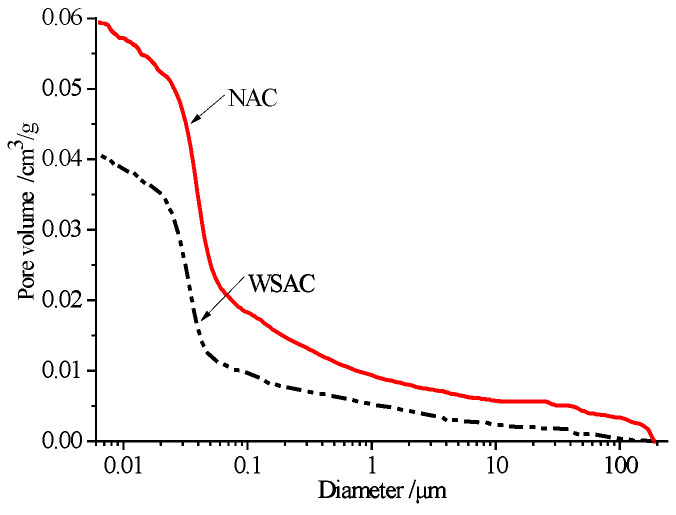
Pore volume of concrete.

**Figure 16:**
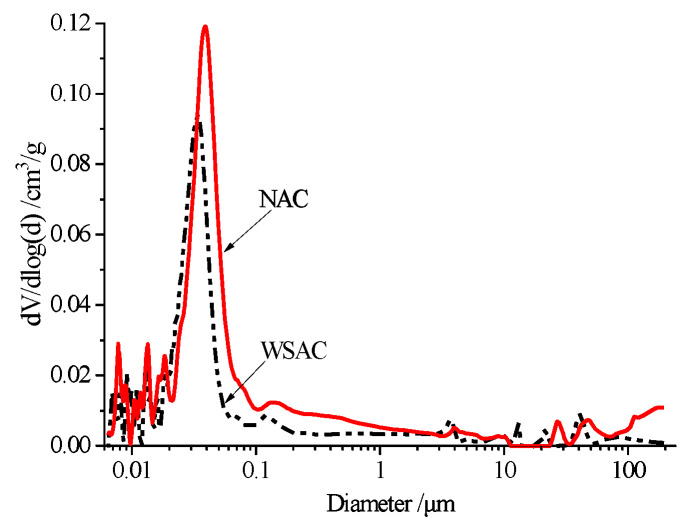
Pore size distributions of concrete.

The pore volume and pore size distribution of the NAC and WSAC samples curing for 120 days are shown in Figure 15 and Figure 16. The porosity of the WSAC sample was lower than NAC’s. The total pore volume of the WASC and NAC specimens were 0.0405 cm^3^/g and 0.0594 cm^3^/g, respectively. The volume percent of the pore below 50 nm was about 76.1% and 69.3% of the WSAC and NAC samples separately according to the pore size distribution results, and the number of the pores larger than 50 nm, which is considered harmful for the durability of concrete, was higher in NAC than WSAC. It is illustrated that the structure of the concrete improved because of the pre-absorbed technology.

### 4.6. Interface of Concrete

**Figure 17:**
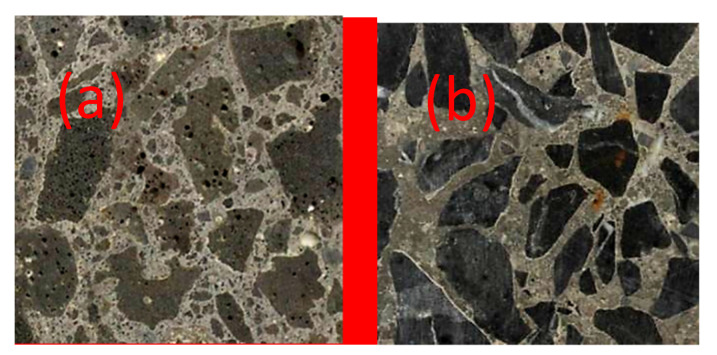
Cross section of concrete. (**a**) with slag aggregate (**b**) with natural aggregate.

The cubic concrete sample was cut to analyze the interface between paste and aggregate, and the results are shown in Figure 17. In Figure 17a the interface between paste and slag aggregate is shown, and the boundary of paste and limestone is shown in Figure 17b. The results show that high titanium heavy slag coarse aggregate is a porous and rough aggregate, internal pores are made up of connected micro-porous and disconnected pores and the surface of aggregate particle has some concave holes. Because of this porous structure, the combination between paste and slag aggregate in WSAC (with 2520 kg/m^3^ dry density) and DSAC (with 2480 kg/m^3^ dry density) was stronger than that of NAC (with 2300 kg/m^3^ dry density).

### 4.7. Stress and Strain Relationship Analysis

**Figure 18:**
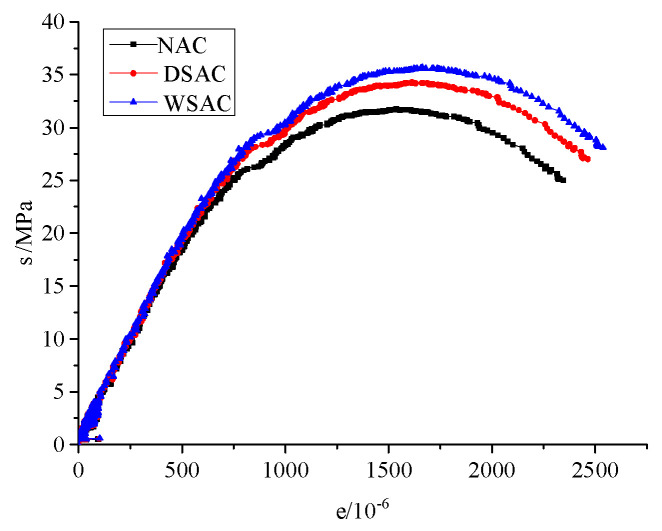
Stress and strain relationships of concrete.

Three samples with each mix were used for the stress and strain relationship analysis. The average stress and strain relationships of concrete with various aggregates are presented in Figure 18. From the results, it is indicated that the maximum stress of the CSAC samples was higher than the others, and the strains of the CSAC and WSAC specimens were larger than that of NAC under peak stress. The slopes of the stress–strain curves before 30% of peak stress of the CSAC and WSAC samples were larger, which illustrates the elastic modulus of concrete with slag aggregate was higher.

**Figure 19:**
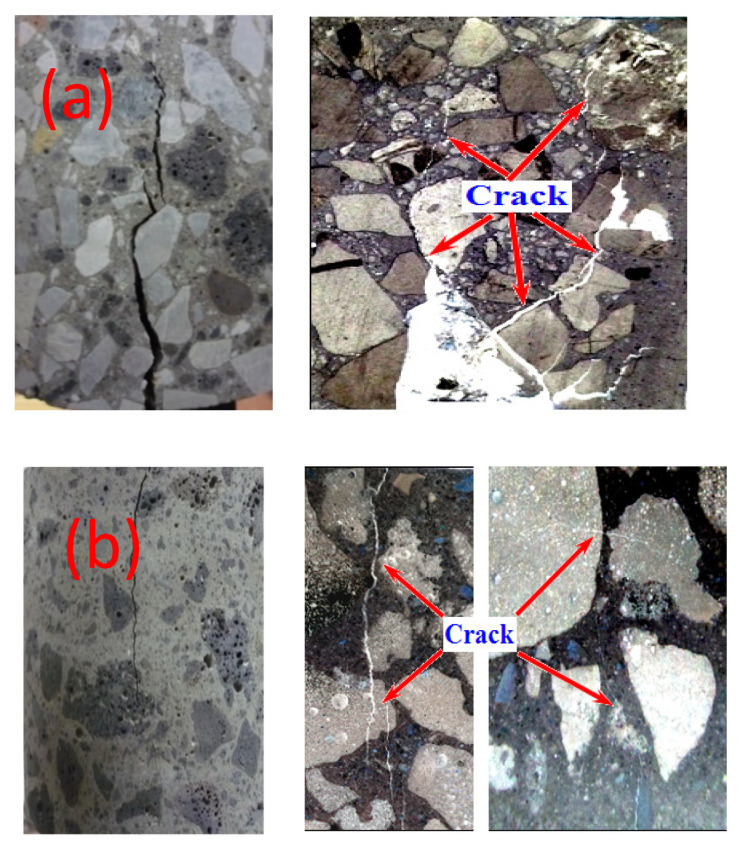
Failure morphology of concrete under compressive loading. (**a**) concrete with natural aggregate (**b**) concrete with slag aggregate.

Figure 19 presents the failure morphology of samples under compressive loading. It is shown that, for concrete with limestone and river sand, the cracks extended along the interface between the aggregate and paste. However, for the concrete with slag aggregate, the cracks spread through the aggregate, which illustrated that the strength of the ITZ in the CSAC and WSAC samples was improved.

### 4.8. Chloride Diffusion Results

**Figure 20:**
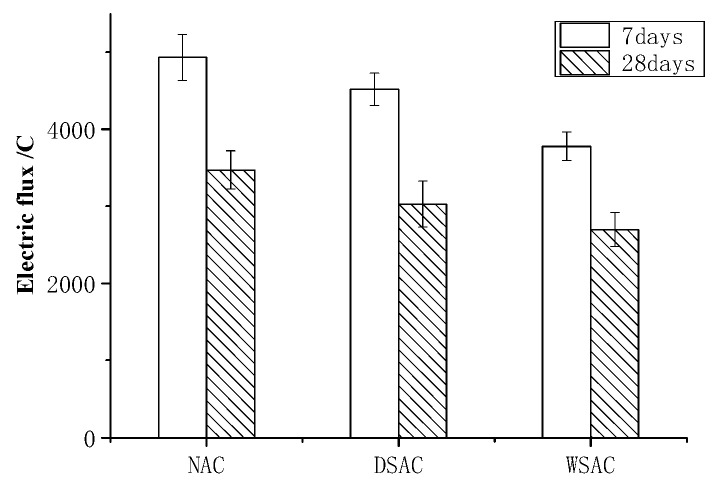
The electric flux of concrete at 7d and 28d.

Three samples with the size of φ100 × 50 mm of each mix were used for chloride diffusion testing. The average electric flux of concrete is shown in Figure 20. It is shown that the electric flux of all the concrete decreased with the development of curing. When the coarse porous aggregate was used as an internal wetting agent, the chloride penetration resistance of concrete was better. This result coincides with the pore structure analysis.

### 4.9. Micro-Hardness Testing Results

**Figure 21:**
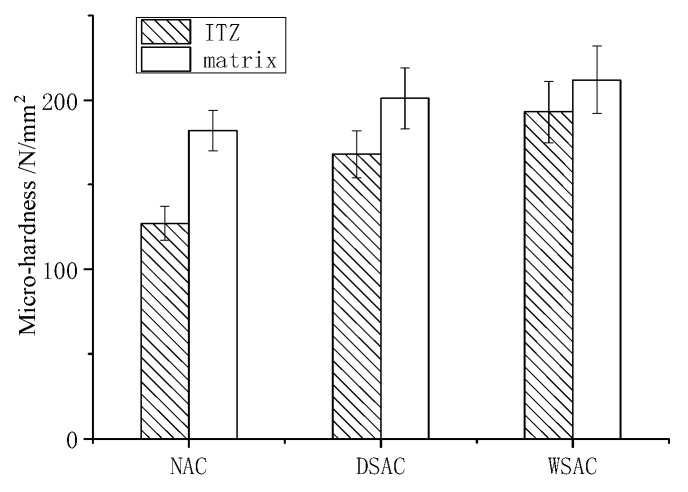
Micro-hardness of ITZ and matrix.

The microhardness of the ITZ and matrix results are displayed in Figure 21. Both the micro-hardness of ITZ and paste matrix of concrete with slag aggregate were obviously higher than that made of natural aggregate. By analyzing the distribution of microhardness in each type of sample, it was found that the ITZ microhardness and matrix hardness ratios were 0.69, 0.83 and 0.91 of NAC, DSAC and WSAC, respectively. The micro-hardness of ITZ and matrix was similar to the WSAC samples, which means the boundary force between the porous aggregate and matrix was stronger.

## 5. Discussion

High titanium heavy slag aggregate, one kind of solid waste, occupies a great deal of land and causes environmental pollution in Guizhou Province in China. Originally, this slag was used as foundation filling material. Preliminary research by our team indicates that there is no harmful and alkali active component in this slag. In this paper, the feasibility of using this slag as an internal curing aggregate in concrete is studied for the porous and rough structure of this kind of slag.

The compressive strength, flexural tensile strength and splitting tensile strength of the samples with dry slag aggregate and pre-wetted slag aggregate were all higher than those of normal aggregate. Additionally, the interfacial micro-hardness testing results show that the interface strength between the paste and slag aggregate was obviously higher than that between paste and limestone because of the surface roughness of the slag. Similarly, the failure morphology of concrete under compressive loading results indicates that the ITZ improved by adding this porous slag aggregate. Furthermore, because of this interface enhancement, the high titanium heavy slag aggregate concrete has better resistance to chloride ion penetration. Therefore, this porous slag can be used as fine and coarse aggregate in concrete.

To make use of this porous structure in slag aggregate, the pre-absorbed technology was adopted in this study. The internal relative humidity and pore size analyses results present that the pre-wetted slag aggregate can play a role as an internal curing agent to increase the relative humidity and decrease the porosity in concrete. Meanwhile, the concrete with pre-wetted slag aggregate can decrease the deformation in drying shrinkage and autogenous shrinkage effectively. Furthermore, using pre-wetted slag aggregate instead of limestone in concrete increases the interface strength in the samples and improves the mechanical properties. Thus, this porous high titanium heavy slag can be used as an internal curing agent in concrete.

## 6. Conclusions

In this paper, the properties of strength, volume stability, interfacial strength and pore structure of NAC, DSAC and WSAC were studied. From the above investigation, the following conclusions can be drawn.

(1) The high titanium heavy slag, one kind of solid waste, can be widely used as an aggregate instead of natural aggregate in concrete. It not only decreases environmental pollution but also improves the strength of concrete.

(2) The high titanium heavy slag is a good internal curing agent in concrete. Concrete composed of pre-wetted coarse slag aggregate and dry fine slag aggregate not only had higher strength and lower dry shrinkage but also had smaller expansion in autogenous shrinkage measurement.

(3) The relative humidity in concrete can be improved by using the slag aggregate as the pre-absorbed material. Furthermore, the porosity and amount of harmful pores decreased because the pre-absorbed water could accelerate the hydration of the cement.

## Figures and Tables

**Table 1:** Chemical compositions of slag aggregate.

Samples	SiO_2_	CaO	TiO_2_	Al_2_O_3_	MgO	Fe_2_O_3_	SO_3_
Fine slag aggregate	30.52	25.76	14.51	14.76	9.09	2.28	0.46
Coarse slag aggregate	26.60	27.75	17.42	13.86	10.76	0.93	0.47
**Samples**	**K_2_O**	**MnO**	**Na_2_O**	**V_2_O_5_**	**BaO**	**SrO**	**P_2_O_5_**
Fine slag aggregate	0.91	0.68	0.50	0.31	0.05	0.03	0.02
Coarse slag aggregate	0.61	0.73	0.40	0.30	0.04	0.03	0.00

**Table 2:** The crush indexes, apparent density and water absorptions of aggregates.

Samples	Crush Index/%	Apparent Density/kg/m^3^	Water Absorption/%
Fine slag aggregate	18.5	3128	8.9
River sand	16.1	2622	4.8
Coarse slag aggregate	14.5	1340	3.38
Limestone	12.1	1480	2.47

**Table 3:** Chemical compositions of cement and fly ash.

Samples	SiO_2_	CaO	TiO_2_	Al_2_O_3_	MgO	Fe_2_O_3_	SO_3_	K_2_O	MnO	Na_2_O	P_2_O_5_	Loss
Cement	22.30	58.40	0.30	5.79	0.91	2.58	2.69	0.82	0.09	0.01	0.10	5.32
Fly ash	48.20	9.60	1.08	26.42	1.23	5.69	0.77	1.30	0.11	0.58	0.29	4.35

**Table 4:** Mix proportion of concrete.

Samples	Water /kg/m^3^	Cement /kg/m^3^	Fly Ash/kg/m^3^	Fine Aggregate/kg/m^3^		Coarse Aggregate/kg/m^3^		Water Reducer /kg/m^3^
				Sand	Fine Slag Aggregate	Limestone	Coarse Slag Aggregate	
NAC	168	300	100	691	0	1037	0	4.0
DSAC	168	300	100	0	765	0	1147	4.0
WSAC	168	300	100	0	765	0	1188	4.0

## Data Availability

Data sharing is not applicable.
